# The interaction between arterial oxygenation and carbon dioxide and hospital mortality following out of hospital cardiac arrest: a cohort study

**DOI:** 10.1186/s13054-020-03039-6

**Published:** 2020-06-12

**Authors:** Peter J. McGuigan, Manu Shankar-Hari, David A. Harrison, John G. Laffey, Danny F. McAuley

**Affiliations:** 1 Regional Intensive Care Unit, Royal Victoria Hospital, Belfast, BT12 6BA UK; 2 Guy’s and St Thomas’ NHS Foundation Trust, ICU support Offices, St Thomas’ Hospital, 1st Floor, East Wing, London, SE1 7EH UK; 3 School of Immunology & Microbial Sciences, Kings College London, London, SE1 9RT UK; 4 Intensive Care National Audit & Research Centre, Napier House, 24 High Holborn, London, WC1V 6AZ UK; 5 Anaesthesia and Intensive Care Medicine, School of Medicine, Regenerative Medicine Institute (REMEDI), CÚRAM Centre for Research in Medical Devices National University of Ireland Galway, Galway, Ireland; 6 Department of Anaesthesia, Galway University Hospitals, Galway, Ireland; 7 Centre for Experimental Medicine, Wellcome-Wolfson Institute for Experimental Medicine, Belfast, BT9 7AE UK

**Keywords:** Out of hospital cardiac arrest, Oxygen, Carbon dioxide, Interaction, Mortality, Critical care

## Abstract

**Background:**

Outcomes following out of hospital cardiac arrest (OHCA) are poor. The optimal arterial oxygen and carbon dioxide (PaCO_2_) levels for managing patients following OHCA are unknown. We hypothesized that abnormalities in arterial oxygenation (PaO_2_/FiO_2_ ratio or PaO_2_) and PaCO_2_ would be associated with hospital mortality following OHCA. We hypothesized that PaCO_2_ would significantly modify the oxygenation–mortality relationship.

**Methods:**

This was an observational cohort study using data from OHCA survivors admitted to adult critical care units in England, Wales and Northern Ireland from 2011 to 2018. Logistic regression analyses were performed to assess the relationship between hospital mortality and oxygenation and PaCO_2_.

**Results:**

The analysis included 23,625 patients. In comparison with patients with a PaO_2_/FiO_2_ > 300 mmHg, those with a PaO_2_/FiO_2_ ≤ 100 mmHg had higher mortality (adjusted OR, 1.79; 95% CI, 1.48 to 2.15; *P* < 0.001). In comparison to hyperoxemia (PaO_2_ > 100 mmHg), patients with hypoxemia (PaO_2_ < 60 mmHg) had higher mortality (adjusted OR, 1.34; 95% CI, 1.10 to 1.65; *P* = 0.004). In comparison with normocapnia, hypercapnia was associated with lower mortality. Hypocapnia (PaCO2 ≤ 35 mmHg) was associated with higher mortality (adjusted OR, 1.91; 95% CI, 1.63 to 2.24; *P* < 0.001). PaCO_2_ modified the PaO_2_/FiO_2_–mortality and PaO_2_–mortality relationships, though these relationships were complex. Patients who were both hyperoxic and hypercapnic had the lowest mortality.

**Conclusions:**

Low PaO_2_/FiO_2_ ratio, hypoxemia and hypocapnia are associated with higher mortality following OHCA. PaCO_2_ modifies the relationship between oxygenation and mortality following OHCA; future studies examining this interaction are required.

## Introduction

In the UK, 30,000 out of hospital cardiac arrests (OHCA) occur each year [1]. Outcomes are poor; of those who survive to ICU admission, just 28.6% survive to hospital discharge [2]. Derangements in oxygenation and carbon dioxide (PaCO_2_) following cardiac arrest (CA) may exacerbate the post-cardiac arrest syndrome [3]. Hence, there has been a recent focus on the management of arterial oxygen and PaCO_2_ in an effort to improve outcomes [4–18]. However, definitions of hypoxemia, hyperoxemia, hypocapnia and hypercapnia vary between studies making it hard to determine thresholds for benefit or harm. Furthermore, studies typically examine the impact of either oxygenation or PaCO_2_ on outcomes; our knowledge of their interaction is limited [18].

Arterial hypoxemia following CA is associated with higher mortality [4–7]. However, previously used criterion for hypoxemia has combined patients with low PaO_2_ and those with abnormal PaO_2_/FiO_2_ ratios making it hard to determine whether absolute hypoxemia or abnormal gas transfer is implicated in the observed increase in mortality. In addition, this approach creates a heterogenous population where patients may have low PaO_2_/FiO_2_ ratios but consistently be exposed to normoxia [4–7].

Conversely, hyperoxemia may exacerbate cellular injury in CA survivors [19]. The evidence surrounding the effect of hyperoxemia is conflicting. Studies have shown an association between hyperoxemia following CA and mortality [4, 5, 8]. Whilst these studies corrected for a variety of cardiac arrest features and physiological parameters, they did not use extensively validated disease-specific scoring systems or illness severity scores. Subsequent studies which used modified Acute Physiology And Chronic Health Evaluation (APACHE) scores to correct for illness severity found no association between mortality and hyperoxemia [6].

Hypocapnia is associated with worse outcomes following CA [6, 13]. The impact of hypercapnia is more uncertain. A PaCO_2_ > 45 mmHg has been associated with improved neurological outcomes [13, 15]. However, higher levels of hypercapnia have been associated with poor neurological outcome or higher mortality [5, 16, 17].

An interdependence exists between ventilation parameters and oxygenation [6]. However, our understanding of how interactions between arterial oxygenation and PaCO_2_ affect outcome following CA is limited and rarely investigated [6, 15, 18]. One prospective observational study found an association between high mean PaCO_2_ and PaO_2_ in the first 24 h following CA and good neurological outcome [15]. A recent pilot study found no difference in biomarkers of cerebral injury in patients randomized to four combinations of normocapnia, hypercapnia, normoxia or hyperoxemia. In this study, elevated regional cerebral oxygen saturation was seen with both hypercapnia and hyperoxia [18]. It is unclear whether a combination of hyperoxia and hypercapnia would overwhelm anti-oxidant systems or improve survival through increased cerebral oxygenation [18]. This is relevant as cerebral injury accounts for two thirds of deaths in patients admitted to ICU following CA [19]. Further understanding of the effects of arterial oxygenation, PaCO_2_ and their interactions in CA survivors would guide future patient management and inform trial design.

### Hypotheses

We hypothesized that abnormalities in arterial oxygenation (either abnormal PaO_2_/FiO_2_ ratio or PaO_2_) and PaCO_2_ would be independently associated with hospital mortality, in adult patients admitted to intensive care units (ICU) following OHCA. We also hypothesized that PaCO_2_ would modify the relationship between oxygenation and mortality.

## Materials and methods

### Data source

The Case Mix Programme (CMP) is the national clinical audit for adult critical care in England, Wales and Northern Ireland; 100% of adult, general intensive care units participated in the CMP [20]. For consecutive admissions, trained data collectors collect 20 physiological parameters from the first 24 h of ICU admission. In addition, 18 non-physiological predictors of mortality are collected including sociodemographic parameters, APACHE II comorbidity status and primary reason for ICU admission [20]. The Case Mix Programme Database (CMPD) does not collect data on cardiac arrest characteristics. Diagnostic data are coded using the Intensive Care National Audit & Research Centre (ICNARC) Coding Method and are available for 99.8% of ICU admissions [21]. Data undergoes validation prior to pooling into the CMPD. Support for the collection and use of these data has been obtained under Section 251 of the National Health Service Act 2006 (approval number: PIAG 2–10(f)/2005).

Arterial blood gas (ABG) data is recorded in the CMPD. The PaO_2_, PaCO_2_, pH and FiO_2_ are recorded from the ABG with the lowest PaO_2_ in the first 24 h after admission to ICU. We used the worst PaO_2_ in the first 24 h which has been shown to be a better discriminator of hospital mortality than other measures of oxygenation in ICU patients [22].

### Study design and population

We undertook retrospective analysis of the ICNARC CMPD for the period 1 January 2011 to 31 December 2018. We included adult patients (≥ 16 years) admitted to general ICUs who had CPR in the 24-h prior to ICU admission. We included OHCA patients only (further details of the CA definitions used are contained in Additional file 1). We excluded unintubated patients. In keeping with other studies, we only included patients who survived beyond 24 h [9, 10, 12, 23, 24]. We excluded patients with admissions following trauma or surgery, readmissions to ICU within the same hospitalization, those with missing hospital mortality outcome and patients without ABG results.

### Statistical analysis

The co-primary exposures were either PaO_2_/FiO_2_ ratio or PaO_2_. The primary outcome was hospital mortality. We considered PaCO_2_ as the main effect modifier in the PaO_2_/FiO_2_–mortality and PaO_2_–mortality relationships. We report unadjusted and adjusted associations between hospital mortality and PaO_2_/FiO_2_ ratio, PaO_2_ and PaCO_2_.

Abnormalities of gas transfer were categorized as follows: PaO_2_/FiO_2_ ratio ≤ 100 mmHg, 101–200 mmHg, 201–300 mmHg, and > 300 mmHg. The reference category was deemed to be PaO_2_/FiO_2_ > 300 mmHg representing patients without abnormal gas transfer [25]. To assess the impact of PaO_2_ on outcome, we selected three categories: PaO_2_ < 60 mmHg (hypoxemia), 60–100 mmHg (normoxia) and > 100 mmHg (hyperoxemia, reflecting exposure to supra-physiological levels of oxygen). Based on previous research which demonstrated a PaO_2_ of 150–200 mmHg to be associated with the lowest hospital mortality following CA, we chose hyperoxemia as the reference category [6].

We selected five PaCO_2_ categories: ≤ 35 mmHg (hypocapnia), 36–45 mmHg (normocapnia) and three hypercapnia categories: 46–50 mmHg, 51–55 mmHg and > 55 mmHg. Due to the harm associated with hypocapnia and the conflicting evidence surrounding the effect of hypercapnia, normocapnia was chosen as the reference category.

Logistic regression models with the following covariates: year of admission, age in deciles, sex, self-reported ethnicity, pre-admission dependency, presence of severe comorbidity as defined using APACHE II score, primary diagnosis categories (sepsis status, acute coronary syndrome, cardiac arrhythmias, other), maximum temperature and lowest glucose in the first 24-h and Acute Physiology Score of the APACHE II score (APS-APII) were used to examine the relationship between hospital mortality and oxygenation and PaCO_2_. We excluded oxygenation, pH and temperature from the APS-APII score, as they were tested as exposures in our study (Supplementary Table 1, Additional file 1); a similar approach has been used in studies examining the effect of oxygenation on outcomes following CA [7].

In logistic regression model 1, we tested the effect of PaO_2_/FiO_2_ ratio categories on hospital mortality. In logistic regression model 2, we tested the effect of PaO_2_ categories on hospital mortality. We performed a number of sensitivity analyses; we tested the effect of varying thresholds of hyperoxemia (PaO_2_ 101–200 mmHg, 201–300 mmHg and > 300 mmHg) on hospital mortality, with PaO_2_ 101–200 mmHg chosen as the reference category [6]. We repeated all the analyses including those who had died within the first 24 h. In all models, we included PaCO_2_ categories to test for interaction.

All logistic regression models were fitted with robust standard errors to account for clustering by ICU and were reported as odds ratios (OR) with 95% confidence intervals (CI). We used complete case analysis, which has been shown to be unbiased in logistic regression under broad assumptions regarding the missing data mechanism [26]. Reported *p* values are two sided, and a *p* value less than 0.05 was considered statistically significant. As a retrospective cohort study, all outcomes were considered hypothesis-generating only and no adjustment was made for multiple comparisons. Continuous data were summarized as mean and standard deviation (SD), where normally distributed, and median and interquartile range, where not. Categorical data were presented as frequency and percentage. All analyses were performed using Stata/SE version 14.2 (StataCorp LP, College Station, TX).

## Results

**Fig. 1:**
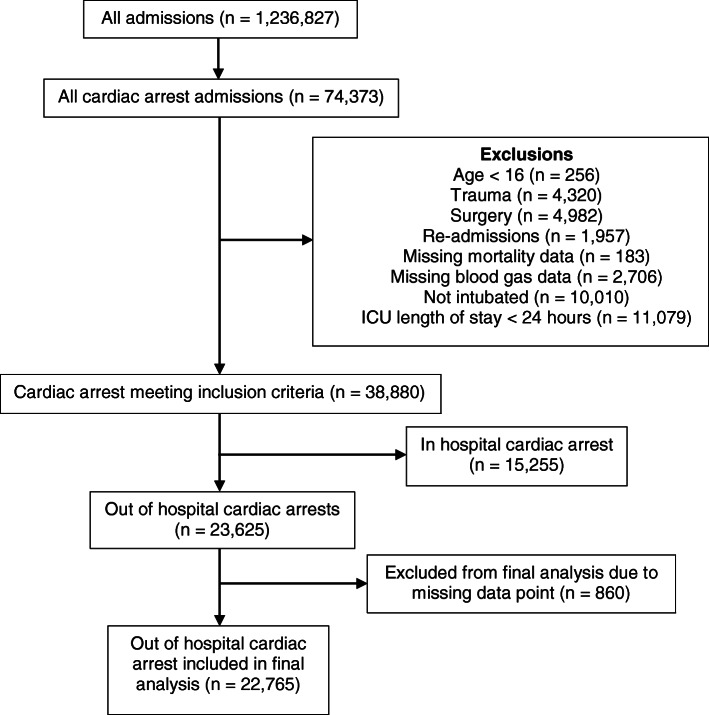
Flow diagram for patient inclusion and exclusion

A total of 74,373 patients admitted following CA were identified; 50,748 met the exclusion criteria (Fig. 1). Patient demographics for 23,625 OHCA patients are shown in Table 1. ICU mortality was 48.9% and hospital mortality 59.1% (Table 1). A total of 860 patients were excluded from the final logistic regression analysis due to missing data. The crude, unadjusted and adjusted odds ratios for hospital mortality are presented in Table 2.

**Table 1:** Patient characteristics

Characteristic	Entire cohort, ***n*** = 23,625
**Male (%)**	16,352 (69.2%)
**Age (years), mean ± SD**	60.6 ± 16.6
**Self-reported ethnicity,*n*(%)**	
White	20,750 ((87.8%)
Mixed	137 (0.6%)
Asian	961 (4.1%)
Black	458 (1.9%)
Other	344 (1.5%)
Not stated	975 (4.1%)
**Pre-admission dependency,*n*(%)**	
Able to live without assistance	18,716 (80.1%)
Some (minor/major) assistance with daily activities	4454 (19.1%)
Total assistance with all daily activities	183 (0.8%)
**Severe co-morbidity**^**a**^**,*n*(%)**	
No	21,469 (90.9%)
Yes	2156 (9.1%)
**APACHE II Acute Physiology Score, mean ± SD**	14.3 ± 6.3
**Primary diagnosis category,*n*(%)**	
Sepsis	998 (4.2%)
Acute coronary syndrome	9166 (38.8%)
Cardiac arrhythmia	9155 (38.8%)
Other	4306 (18.2%)
**FiO**_**2**_**(from ABG with lowest PaO**_**2**_**), median (IQR)** (data missing on *n* = 1)	0.35 (0.3–0.5)
**Central temperature (°C), mean ± SD**	
Maximum temperature in first 24 h (data missing on *n* = 1)	36.7 ± 1.3 °C
Minimum temperature in first 24 h (data missing on *n* = 1)	34.0 ± 1.6 °C
**Glucose (mmol/L), mean ± SD**	
Highest glucose in first 24 h (data missing on *n* = 1926)^b^	12.0 ± 5.2
Lowest glucose in first 24 h (data missing on *n* = 591)	6.5 ± 2.5
**Treatment withdrawal after 24 h (%)**	
No	14,820 (62.7%)
Yes	8805 (37.3%)
**ICU outcome,*n*(%)**	
Survived	12,064 (51.1%)
Died	11,561 (48.9%)
**ICU length of stay (days), median (IQR)**	
All patients	4.0 (2.4–7.1)
ICU survivors	5.2 (3.0–9.9)
ICU non-survivors	3.2 (1.9–5.1)
**Hospital outcome,*n*(%)**	
Survived	9649 (40.8%)
Died	13,976 (59.1%)
**Hospital length of stay (days), median (IQR)**	
All patients	7 (3–17)
Hospital survivors	19 (11–34)
Hospital non-survivors	4 (2–7)

^a^See Supplementary Table 1, Additional file 1. For definitions of APACHE II severe co-morbidities

^b^In patients where only one glucose is recorded, ICNARC CMPD records this as the lowest glucose; this results in a greater number of patients with missing highest glucose values

**Table 2:** Impact of PaO_2_/FiO_2_, PaO_2_ and PaCO_2_ on hospital mortality

Variable	Incidence, ***n*** (%)	Hospital mortality, ***n*** (%)	Unadjusted odds ratio for hospital mortality (95% CI)	Adjusted odds ratio for hospital mortality (95% CI)
PaO_2_/FiO_2_^a^				
≤ 100 mmHg	3314/23,624 (14.0%)	2268/3314 (68.4%)	2.18 (1.98–2.39), *P* < 0.001	1.79 (1.48–2.15), *P* < 0.001
101–200 mmHg	8393/23,624 (35.5%)	5304/8393 (63.2%)	1.72 (1.60–1.85), *P* < 0.001	1.63 (1.45–1.84), *P* < 0.001
201–300 mmHg	7313/23,624 (31.0%)	4105/7313 (56.1%)	1.28 (1.19–1.38), *P* < 0.001	1.36 (1.21–1.53), *P* < 0.001
> 300 mmHg	4604/23,624 (19.5%)	2298/4604 (49.9%)	1 (Reference category)	1 (Reference category)
PaO_2_^b^				
Hypoxemia	4135/23,625 (17.5%)	2704/4135 (65.4%)	1.61 (1.45–1.80), *P* < 0.001	1.34 (1.10–1.65), *P* = 0.004
Normoxia	17,480/23,625 (74.0%)	10,187 / 17,480 (58.3%)	1.19 (1.09–1.31), *P* < 1.001	1.15 (0.98–1.35), *P* = 0.082
Hyperoxemia	2010/23,625 (8.5%)	1085/2010 (54.0%)	1 (Reference category)	1 (Reference category)
PaCO_2_^a^				
≤ 35 mmHg	5554/23,625 (23.5%)	3621/5554 (65.2%)	1.44 (1.34–1.54), *P* < 0.001	1.91 (1.63–2.24), *P* < 0.001
36–45 mmHg	9910/23,625 (41.9%)	5605/9910 (56.6%)	1 (Reference category)	1 (Reference category)
46–50 mmHg	3444/23,625 (14.6%)	1906/3444 (55.3%)	0.95 (0.88–1.03), *P* = 0.215	0.69 (0.55–0.86), *P* = 0.001
51–55 mmHg	1958/23,625 (8.3%)	1079/1958 (55.1%)	0.94 (0.86–1.04), *P* = 0.237	0.74 (0.53–1.04), *P* = 0.079
> 55 mmHg	2759/23,625 (11.7%)	1765/2759 (64.0%)	1.36 (1.25–1.49), *P* < 0.001	0.40 (0.23–0.70), *P* = 0.001

^a^Reported using logistic regression model 1, data missing on *n* = 1

^b^Reported using logistic regression model 2

### PaO_2_/FiO_2_–mortality relationship

The majority of patients (80.5%) had abnormal gas transfer. Crude hospital mortality was highest in those with PaO_2_/FiO_2_ ≤ 100 mmHg (68.4%). Worsening PaO_2_/FiO_2_ ratios were associated with higher mortality (all *P* < 0.001). Patients with a PaO_2_/FiO_2_ ≤ 100 mmHg had an almost twofold higher mortality than those with a PaO_2_/FiO_2_ ratio > 300 mmHg (Table 2).

### PaO_2_–mortality relationship

In comparison to the hyperoxemia group, the hypoxic group had a significantly higher mortality. There was no difference in mortality between normoxia and hyperoxemia (Table 2).

### PaCO_2_–mortality relationship

**Fig. 2:**
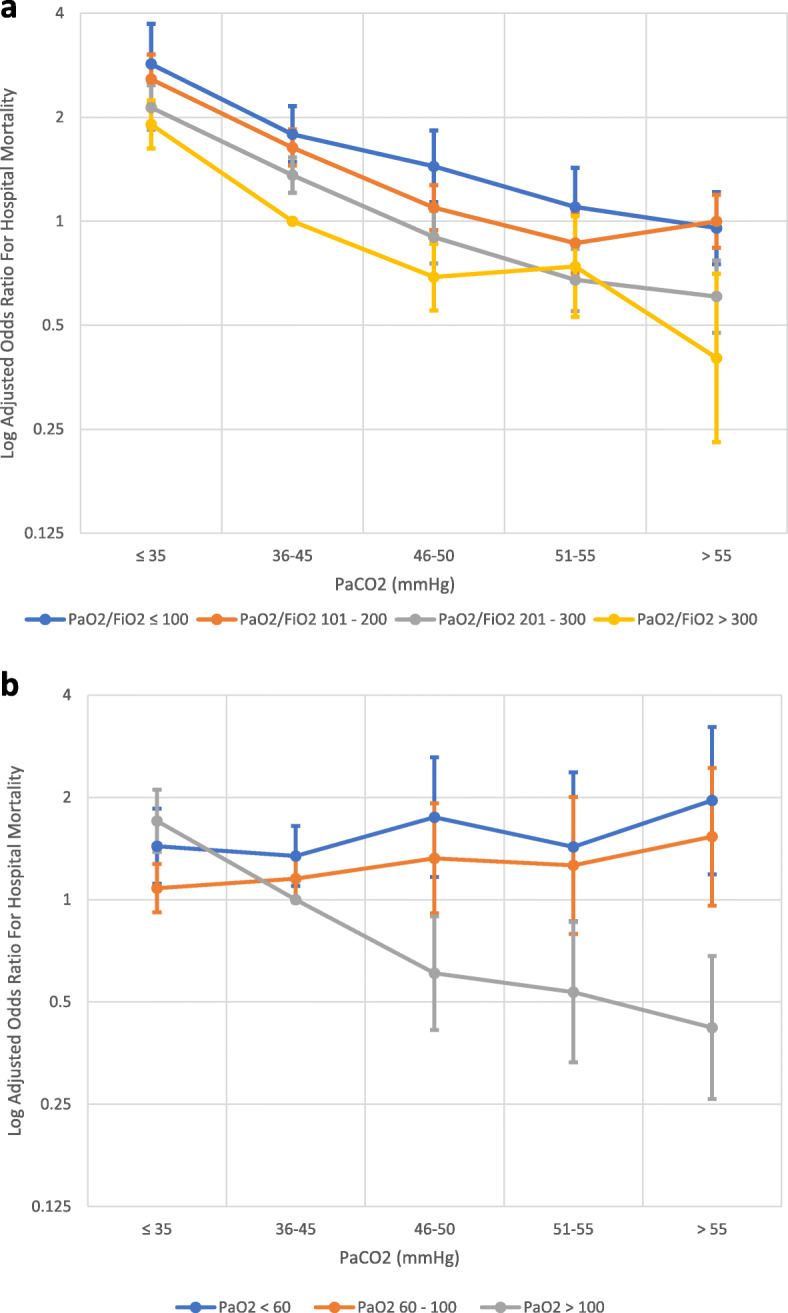
**a** Adjusted odds ratio for mortality PaO_2_/FiO_2_ versus PaCO_2_ derived using logistic regression model 1. Presented using a semi-logarithmic scale. **b** Adjusted odds ratio for mortality PaO_2_ versus PaCO_2_ derived using logistic regression model 2. Presented using a semi-logarithmic scale

**Fig. 3:**
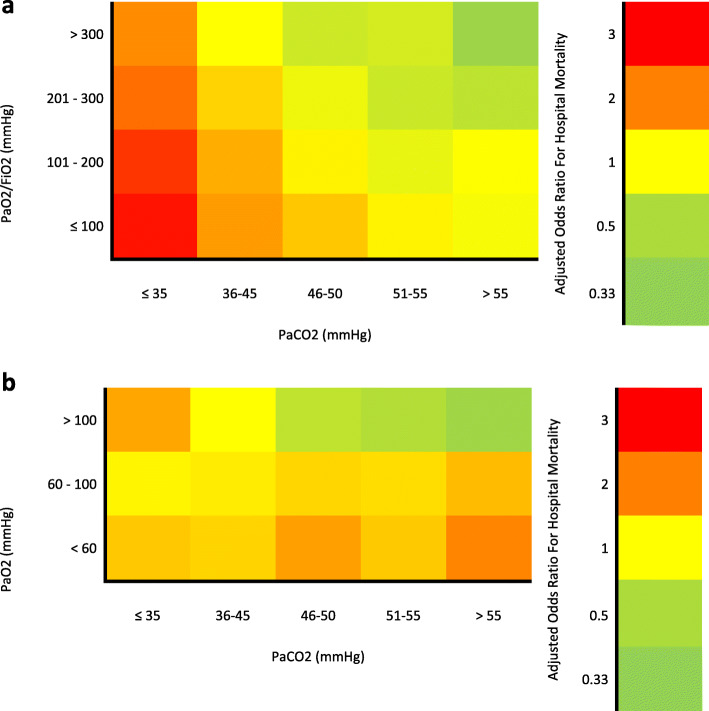
**a** Impact of PaCO_2_ on PaO_2_/FiO_2_–mortality relationship. Heat map demonstrating how PaCO_2_ modifies the PaO_2_/FiO_2_–mortality relationship. **b** Impact of PaCO_2_ on PaO_2_–mortality relationship. Heat map demonstrating how PaCO_2_ modifies the PaO_2_–mortality relationship

In the unadjusted analysis, the relationship between PaCO_2_ and hospital mortality was U-shaped (Table 2). In the adjusted analysis, hypocapnia was associated with higher hospital mortality, whereas a PaCO_2_ of 46–50 mmHg and > 55 mmHg was associated with lower hospital mortality (Table 2). PaCO_2_ modified the PaO_2_/FiO_2_–mortality and PaO2–mortality relationships (Figs. 2, 3, Supplementary Table 3 and 4, Additional file 1).

### The impact of hypocapnia on the PaO_2_/FiO_2_–mortality relationship

In all PaO_2_/FiO_2_ categories, the presence of hypocapnia was associated with higher mortality when compared with normocapnia (Supplementary Table 3, Additional file 1).

### The impact of hypocapnia on the PaO_2_–mortality relationship

In patients with hypoxemia and normoxia, hypocapnia did not demonstrate a consistent effect on mortality when compared to normocapnia. In contrast, in patients with hyperoxemia, the presence of hypocapnia was associated with higher mortality when compared to normocapnia (Supplementary Table 4, Additional file 1).

### The impact of hypercapnia on the PaO_2_/FiO_2_–mortality relationship

In all PaO_2_/FiO_2_ categories, hypercapnia was repeatedly associated with lower mortality. In the PaO_2_/FiO_2_ analysis, the lowest mortality was observed in those with a PaO_2_/FiO_2_ > 300 mmHg and a PaCO2 > 55 mmHg (adjusted OR, 0.40; 95% CI 0.23 to 0.70; *P* = 0.001) (Supplementary Table 3, Additional file 1).

### The impact of hypercapnia on the PaO_2_–mortality relationship

In patients with hypoxemia or normoxia, those with hypercapnia had a higher mortality when compared to those with normocapnia. In contrast, in those with hyperoxemia, all categories of hypercapnia were associated with significantly lower mortality which followed a dose-dependent pattern: PaCO_2_ 46–50 mmHg (adjusted OR, 0.61; 95% CI, 0.41 to 0.89; *P* = 0.011), PaCO_2_ 51–55 mmHg (adjusted OR, 0.53; 95% CI, 0.33 to 0.86; *P* = 0.010) and PaCO_2_ > 55 mmHg (adjusted OR, 0.42; 95% CI, 0.26 to 0.63; *P* < 0.001). The lowest mortality overall was observed in patients with hyperoxemia and PaCO_2_ > 55 mmHg (Supplementary Table 4, Additional file 1).

### Sensitivity analysis

The results of the sensitivity analyses are presented in Supplementary Tables 5 to 11, Additional file 1. In the PaO_2_ five-category model, the number of patients with a PaO_2_ 201–300 mmHg or PaO_2_ > 300 mmHg was small (*n* = 40 and *n* = 31, respectively). There was no statistically significant difference in mortality between a PaO_2_ 201–300 mmHg or PaO_2_ > 300 mmHg and the reference category of PaO_2_ 101–200 mmHg (Supplementary Table 5 and 6, Additional file 1).

Sensitivity analyses including patients who died within the first 24 h were performed.

Crude, adjusted and unadjusted analysis produced near identical results to the main analysis (Supplementary Tables 7–9, Additional file 1). However, when those who died within the first 24 h were included, a PaO_2_ > 300 mmHg was associated with higher mortality (Supplementary Table 10 and 11, Additional file 1).

### Analysis of the performance of the logistic regression model

Of the components included in the logistic regression analysis (Supplementary Table 1, Additional file 1), pH had the greatest influence on outcome, with acidosis being associated with increased mortality (Supplementary Table 2, Additional file 1). Variance inflation factors indicated there was no co-linearity between any of the ABG values used in our analysis.

## Discussion

In this large retrospective study of patients admitted to ICU following OHCA, we found a significant association between hypoxemia and worsening PaO_2_/FiO_2_ ratios and mortality. This is in keeping with the other large CA databases [4–7, 12]. This has biological rationale as hypoxemia is a marker for pulmonary pathology and exacerbates myocardial dysfunction and cerebral injury [27]. No association between hyperoxemia and mortality was observed. Importantly, we found PaCO_2_ modified the PaO_2_/FiO_2_–mortality and PaO_2_–mortality relationships.

The post-cardiac arrest syndrome is characterized by a widespread ischaemia/reperfusion response [28]. Post-ischaemic tissue is susceptible to oxygen free radical damage, resulting in reduced left ventricular function, coronary artery vasoconstriction and myocardial ischaemia [29]. Hyperoxemia may exacerbate oxygen free radical-mediated damage in the brain and promote pulmonary inflammation [4, 30]. In contrast, a recent porcine model of CA demonstrated a reduced incidence of low brain tissue oxygenation in swine treated with a FiO_2_ of 1.0 and a 20-mmHg increase in mean arterial pressure (MAP) from baseline compared to those treated with a SpO2 target of 94–98% and a MAP target of > 65 mmHg [31]. This may help explain our finding of a lower mortality associated with hyperoxia. Previous studies which demonstrated an association between hyperoxemia and mortality following CA did not use validated scores to correct for illness severity [4, 5]. Our study, derived from a high-quality database, demonstrated no association between mortality and hyperoxemia or PaO_2_/FiO_2_ > 300 mmHg and is in keeping with similar studies [6]. Indeed, we found patients with hyperoxemia to have the lowest mortality. A sensitivity analysis examining different thresholds of hyperoxia found no association between hyperoxia and mortality. Following CA, the risk of exposure to hyperoxia falls with time, hence the low number of patients with a lowest PaO_2_ > 300 mmHg [10]. However, the risk of a type II error is high. In sensitivity analyses including those who died within the first 24 h, a PaO_2_ > 300 mmHg was associated with a higher mortality. Two thirds of patients who do not survive the first 24 h following OHCA have withdrawal of life-sustaining therapy based on pre-existing co-morbidities or perceived poor neurological prognosis [32]. Thus, for the majority of deaths within the first 24 h, the risk of mortality is unrelated to exposure to oxygenation or PaCO_2_. As the risk of exposure to hyperoxia falls with time during the first 24 h following OHCA [10], those who die within the first 24 h have a disproportionate risk of having a lowest PaO_2_ in the hyperoxia range. In our study, these patients were excluded to avoid this important potential confounding variable.

In keeping with other studies, we found hypercapnia to be associated with lower mortality [13–15]. Hypercapnia modified both the PaO_2_/FiO_2_–mortality and PaO_2_–mortality relationships. The mortality benefit was seen in all PaO_2_/FiO_2_ categories but was confined to hyperoxic patients in the PaO_2_–mortality model.

Following CA, cerebral vasoconstriction and a loss of cerebral autoregulation have been demonstrated [33, 34]. Hypercapnia may increase cerebral blood flow, improve cerebral oxygenation, exhibit direct neuroprotective effects, reduce pulmonary and systemic inflammation and reduce oxygen free radical-mediated tissue injury [13, 15, 35–40]. In CA survivors, hypercapnia may attenuate oxygen free radical production. Alternatively, the observed benefit of hypercapnia over normocapnia may be attributable to injurious ventilation strategies used to achieve normocapnia [3].

Hypercapnia was repeatedly associated with lower mortality in the PaO_2_/FiO_2_ model. However, hypercapnia was not associated with lower mortality in the setting of hypoxemia or normoxia in the PaO_2_–mortality model. This may represent a sick cohort of patients with respiratory failure and poor pulmonary compliance not fully corrected for in the PaO_2_–mortality model [41]. Hypercapnic acidosis causes pulmonary hypertension, right ventricular strain, reduced coronary blood flow and cerebral oedema [42, 43]. These effects are accentuated by hypoxemia [44, 45].

Hypocapnia was associated with higher mortality in all categories in the PaO_2_/FiO_2_–mortality model and in patients with hyperoxemia. Hyperoxemia and hypocapnia both cause cerebral vasoconstriction reducing cerebral blood flow [46–48]. Hypocapnia shifts the oxygen dissociation curve impairing oxygen delivery [47]. Together, these may exacerbate cerebral ischaemia.

The ability of PaCO_2_ to modify outcomes depended on whether a PaO_2_/FiO_2_ or PaO_2_ model was used. In addition, PaCO_2_ had limited influence on outcomes in patients with hypoxemia and normoxia. It is likely that hypoxemia is the overwhelming factor determining mortality and PaCO_2_ has a limited ability to modify this outcome. However, our understanding of the pathophysiology of the modifying impact of PaCO_2_ is poor.

Our observational study has a number of strengths. We investigated the association between PaO_2_/FiO_2_ and absolute PaO_2_ on mortality separately and unlike in other studies tested for interaction with PaCO_2_. The cohort was larger than other studies in this area [4–18, 42]. Thus, allowing us to treat PaCO_2_ as a categorical variable and investigate whether a threshold existed beyond which hypercapnia became harmful. Our cohort was derived from a high-quality database allowing correction for confounding variables. Our findings are supported by sensitivity analyses. We used the Acute Physiology Score component of the APACHE II score to correct for illness severity having excluded oxygenation, pH and temperature as they were tested as primary exposures in our model. The Acute Physiology Score has previously been demonstrated to have a better positive predictive value in predicting mortality following cardiac arrest than the APACHE II score [49]. The use of modified APACHE scores to correct for illness severity when examining the association between oxygenation and carbon dioxide and outcomes following cardiac arrest is well established [6, 7, 13, 15] but not universally applied [4, 5, 7, 9–11, 16, 17]. The APACHE II score has previously been shown to have a similar ability to predict mortality following OHCA as the disease-specific OHCA score [50].

Our study has a number of limitations. As a cohort study, causality cannot be inferred. It is possible that residual confounders remain. No data was available on intra-arrest characteristics. Hence, we have been unable to account for presenting rhythm, bystander CPR or defibrillation or duration of delay to ROSC, all of which significantly impact on patient outcomes [51]. For OHCA patients, APACHE III scores showed a modest ability to predict mortality, whereas delay to ROSC showed a good ability to predict mortality [52]. We acknowledge that intra-arrest characteristics, including the delay to ROSC, are better predictors of outcome following cardiac arrest than illness severity scores. Unfortunately, no such data was collected within the ICNARC-CMPD. During our study period, there may have been temporal changes in cardiac arrest management including temperature control post-cardiac arrest; to account for this, we adjusted for year of admission [53]. Our outcome measure was hospital mortality; no data was available on longer-term mortality or neurological outcomes.

Previous studies have demonstrated an association between hyperoxemia on admission to ICU and mortality following CA [4, 5]. We cannot exclude that exposure to derangements in oxygenation and PaCO_2_ in the immediate post-ROSC period is more prognostically significant than derangements as recorded in the ICNARC database. However, this is unlikely, as the worst PaO_2_ in the first 24 h predicts ICU mortality more accurately than PaO_2_ on the first ABG in a general ICU population [22]. Additionally, cumulative exposure to hyperoxemia over the first 24 h following CA has also been associated with mortality; the ICNARC-recorded ABG provides a surrogate for cumulative oxygen exposure during the first 24 h [10].

It could be argued that the ABG data collected may not be truly representative of an individual’s exposure to derangements in oxygenation following CA. However, the ICNARC-recorded ABG uses methods similar to the APACHE methodology. The PaO_2_ recorded using the APACHE methodology is more representative of the mean PaO_2_ in the first 24, 48 and 72 h following CA than an ABG taken on admission to ICU [4, 7]. A further limitation is the use of the ICNARC-recorded PaCO_2_; however, the PaCO_2_ recorded using similar APACHE methodology correlates closely with PaCO_2_ in the first 24 h in CA survivors [13].

In examining PaO_2_/FiO_2_ and PaO_2_ separately, we have demonstrated that derangements in both were associated with higher mortality [4–7]. Despite a large number of patients with abnormal gas transfer, patients were typically normoxic; this may reflect titration of FiO_2_ and adherence to ILCOR guidelines [3, 54]. However, it may have contributed to the different behaviour of the PaO_2_/FiO_2_ and PaO_2_ models in relation to the modifying impact of PaCO_2_. In our PaO_2_/FiO_2_ model, we were unable to differentiate between low PaO_2_/FiO_2_ ratios due to ARDS, pulmonary oedema or pre-existing lung pathology. Whilst we corrected for the presence of APACHE II-defined severe respiratory co-morbidity, residual confounding due to pre-existing respiratory pathology may have remained.

Finally, in choosing PaO_2_ > 100 mmHg as the threshold for hyperoxemia, we may have missed harm associated with a higher threshold [55]. To address this, we presented a sensitivity analysis. There are several reasons to justify our threshold of 100 mmHg; a PaO_2_ > 100 mmHg is rarely observed in health (hence, patients where the lowest PaO_2_ is > 100 mmHg have been exposed to supraphysiological levels of oxygenation over a 24-h period and are by definition hyperoxemic), a PaO_2_ of 150-200 mmHg has been associated with the lowest hospital mortality in a post-cardiac arrest population, a similar threshold has previously been used when investigating hyperoxemia in a general ICU population and choosing this threshold identified a large hyperoxic patient cohort reducing the risk of a type II error [6, 32]. However, conclusions from our sensitivity analysis are limited by the small number of patients in the hyperoxemia categories, resulting in a risk of a type II error.

## Conclusions

We found an association between hypoxemia, low PaO_2_/FiO_2_ ratios and hypocapnia and mortality following OHCA. As hypothesized, PaCO_2_ modifies the relationship between oxygenation and mortality, though the relationships are complex. Our study emphasizes the need for future studies examining the interaction of PaO_2_ and PaCO_2_ in OHCA survivors.

## Supplementary information

**Additional file 1: **The interaction between arterial oxygenation and carbon dioxide and hospital mortality following out of hospital cardiac arrest; a cohort study. Provides details on variables used in the logistic regression analysis and of how PaCO_2_ modified the PaO_2_/FiO_2_ – mortality and PaO_2_ – mortality relationships. Sensitivity analyses are presented examining the effects of varying thresholds of hyperoxia on mortality and the effect of including those who died in the first 24 hours. **Table 1** Variables used in logistic regression analysis Model-1 and Model-2. ^a^APACHE II severe co-morbidities include the following: 1. Liver: biopsy proven cirrhosis with portal hypertension, previous upper gastrointestinal bleeding secondary to portal hypertension, previous hepatic encephalopathy or failure. 2. Cardiovascular: New York Heart Association Classification IV. 3. Respiratory: pulmonary disease resulting in severe exercise limitation, chronic hypoxemia or hypercapnia, severe pulmonary hypertension or ventilator dependency. 4. Renal: Dialysis dependent renal failure. 5. Immunocompromised: either drug or disease induced. **Table** 2 Odds ratio for mortality for six pH categories used in logistic regression analysis Model-1. Table 3 Adjusted odds ratio for mortality PaO_2_/FiO_2_ versus PaCO_2_ derived using logistic regression Model-1. Table 4 Adjusted odds ratio for mortality PaO_2_ versus PaCO_2_ derived using logistic regression Model-2. Table 5 Sensitivity analysis; Impact of varying thresholds of hyperoxia on hospital mortality. Five PaO_2_ categories were chosen to examine the impact of alternative thresholds of hyperoxemia on mortality. **Table 6** Sensitivity analysis; Adjusted odds ratio for mortality for varying thresholds of PaO_2_ versus PaCO_2_. Five PaO_2_ categories were chosen to examine the impact of alternative thresholds of hyperoxemia on mortality, PaCO_2_ categories were included to test for interaction. ^a^Due to the small number of patients in this subcategory of the sensitivity analysis (all of whom died), the interaction term has been dropped from the model and the category therefore has the same OR for mortality as the reference category. **Table 7** Sensitivity analysis; Impact of PaO_2_/FiO_2_, PaO_2_ and PaCO_2_ on hospital mortality including those who died within the first 24 hours. **Table 8** Sensitivity analysis; Adjusted odds ratio for mortality PaO_2_/FiO_2_ versus PaCO_2_ including those who died within the first 24 hours. **Table 9** Sensitivity analysis; Adjusted odds ratio for mortality PaO_2_ versus PaCO_2_ including those who died within the first 24 hours. Here, the ability of PaCO_2_ to modify mortality relationships is attenuated. **Table 10** Sensitivity analysis; Impact of varying thresholds of hyperoxia on hospital mortality including those who died within the first 24 hours. Five PaO_2_ categories were chosen to examine the impact of alternative thresholds of hyperoxemia on mortality including those who died within the first 24 hours. **Table 11** Sensitivity analysis; Adjusted odds ratio for mortality for varying thresholds of PaO_2_ versus PaCO_2_ including those who died within the first 24 hours. Here, the ability of PaCO_2_ to modify mortality relationships is attenuated. ^a^Due to the small number of patients in this subcategory of the sensitivity analysis (all of whom died), the interaction term has been dropped from the model and the category therefore has the same OR for mortality as the reference category.

## Data Availability

The data that support the findings of this study are available from ICNARC, but restrictions apply to the availability of these data, which were used under license for the current study, and so are not publicly available. Data are however available from the authors upon reasonable request and with permission of ICNARC’s independent Data Access Advisory Group.

## Abbreviations

ABG: Arterial blood gas
APACHE: Acute physiology and chronic health evaluation
APS-APII: Acute physiology score of acute physiology and chronic health evaluation II score
CA: Cardiac arrest
CI: Confidence intervals
CMPD: Case Mix Programme database
CPR: Cardiopulmonary resuscitation
ICNARC: Intensive Care National Audit & Research Centre
ICU: Intensive care units
IHCA: In-hospital cardiac arrest
ILCOR: International Liaison Committee on Resuscitation
OHCA: Out of hospital cardiac arrests
OR: Odds ratio
PaCO_2_: Arterial partial pressure of carbon dioxide
PaO_2_: Arterial partial pressure of oxygen
PaO_2_/FiO_2_: Ratio of arterial partial pressure of oxygen divided by fraction of inspired oxygen
ROSC: Return of spontaneous circulation
