# Supplementary material for: The interaction between arterial oxygenation and carbon dioxide and hospital mortality following out of hospital cardiac arrest: a cohort study
Source: Crit Care. 2020 Jun 12;24:336. doi: 10.1186/s13054-020-03039-6 (PMC7290139; doi:10.1186/s13054-020-03039-6)
Supplement: Supplementary file 1 — Additional file 1: The interaction between arterial oxygenation and carbon dioxide and hospital mortality following out of hospital cardiac arrest; a cohort study. Provides details on variables used in the logistic regression analysis and of how PaCO2 modified the PaO2/FiO2 – mortality and PaO2 – mortality relationships. Sensitivity analyses are presented examining the effects of varying thresholds of hyperoxia on mortality and the effect of including those who died in the first 24 hours. Table 1 Variables used in logistic regression analysis Model-1 and Model-2. aAPACHE II severe co-morbidities include the following: 1. Liver: biopsy proven cirrhosis with portal hypertension, previous upper gastrointestinal bleeding secondary to portal hypertension, previous hepatic encephalopathy or failure. 2. Cardiovascular: New York Heart Association Classification IV. 3. Respiratory: pulmonary disease resulting in severe exercise limitation, chronic hypoxemia or hypercapnia, severe pulmonary hypertension or ventilator dependency. 4. Renal: Dialysis dependent renal failure. 5. Immunocompromised: either drug or disease induced. Table 2 Odds ratio for mortality for six pH categories used in logistic regression analysis Model-1. Table 3 Adjusted odds ratio for mortality PaO2/FiO2 versus PaCO2 derived using logistic regression Model-1. Table 4 Adjusted odds ratio for mortality PaO2 versus PaCO2 derived using logistic regression Model-2. Table 5 Sensitivity analysis; Impact of varying thresholds of hyperoxia on hospital mortality. Five PaO2 categories were chosen to examine the impact of alternative thresholds of hyperoxemia on mortality. Table 6 Sensitivity analysis; Adjusted odds ratio for mortality for varying thresholds of PaO2 versus PaCO2. Five PaO2 categories were chosen to examine the impact of alternative thresholds of hyperoxemia on mortality, PaCO2 categories were included to test for interaction. aDue to the small number of patients in this subcategory of the sensitivity [file 13054_2020_3039_MOESM1_ESM.doc]

**The interaction between arterial oxygenation and carbon dioxide and hospital mortality following out of hospital cardiac arrest; a retrospective cohort study.**

**Supplemental digital content**

**Cardiac arrest definitions**

Patients were defined as having an OHCA when the location immediately prior to ICU admission was ‘clinic or home’ and source of admission to the ICU was ‘A&E in the same hospital’. Patients were defined as having an IHCA when the location immediately prior to ICU admission was not ‘clinic or home’ and source of admission to the ICU was not ‘A&E in the same hospital’.

**Table 1**

| Variable |  |  |  |  |  |  |
| --- | --- | --- | --- | --- | --- | --- |
| PaO_2_/FiO_2_ (mmHg) | ≤ 100 | 101 - 200 | 201 - 300 | > 300 |  |  |
| PaO_2_ (mmHg) | < 60 | 60 - 100 | > 100 |  |  |  |
| PaCO_2_ (mmHg) | ≤ 35 | 36 - 45 | 46 - 50 | 51 - 55 | > 55 |  |
| Year of admission | Categorical variable in years | | | | | |
| APACHE II Acute Physiology Score | Continuous variable.  Excluding PaO_2_/FiO_2_, pH and temperature components. | | | | | |
| pH | < 6.90 | 6.91 - 7.149 | 7.15 - 7.249 | 7.25 - 7.349 | 7.35 - 7.45 | > 7.451 |
| Dependency  category | Able to live without assistance | Minor / major assistance with daily activities | Total assistance with all daily activities |  |  |  |
| Sex | Male | Female |  |  |  |  |
| Age | Categorical variable.  Age in deciles | | | | | |
| Self-reported ethnicity | White | Mixed | Asian | Black |  |  |
| Severe co-morbidity*^a^* | Yes | No |  |  |  |  |
| Primary diagnosis category | Sepsis | Acute Coronary Syndrome | Cardiac arrhythmia | Other |  |  |
| Maximum Central temperature (^o^C) | Continuous variable.  Maximum central temperature in first 24 hours. | | | | | |
| Minimum Central temperature (^o^C) | Continuous variable.  Minimum central temperature in first 24 hours. | | | | | |
| Maximum glucose (mmol/L) | Maximum glucose in first 24 hours | | | | | |
| Minimum glucose (mmol/L) | Minimum glucose in first 24 hours | | | | | |
| Treatment withdrawal after 24 hours | Yes | No |  |  |  |  |

**Table 2**

| pH | OR for mortality |
| --- | --- |
| Min - 6.90 | 2.93 (1.96 – 4.38), P < 0.001 |
| 6.91 - 7.149 | 2.96 (2.60 – 3.38), P < 0.001 |
| 7.15 - 7.249 | 2.35 (2.18 – 2.54), P < 0.001 |
| 7.25 - 7.349 | 1.51 (1.43 – 1.60), P < 0.001 |
| 7.35 - 7.45 | 1 (reference category) |
| 7.451 - max | 0.93 (0.85 – 1.02), P = 0.135 |

**Table 3**

|  | | PaO_2_/FiO_2_ | | | |
| --- | --- | --- | --- | --- | --- |
|  |  | ≤ 100mmHg  AOR (95% CI) | 101 - 200mmHg  AOR (95% CI) | 201 - 300mmHg  AOR (95% CI) | > 300mmHg  AOR (95% CI) |
| PaCO_2_ | ≤ 35mmHg | 2.85 (2.18 - 3.73) | 2.58 (2.19 - 3.04) | 2.13 (1.84 - 2.48) | 1.91 (1.63 - 2.24) |
|  | 36 - 45mmHg | 1.79 (1.48 - 2.15) | 1.63 (1.45 - 1.84) | 1.36 (1.21 - 1.53) | 1 (reference category) |
|  | 46 - 50mmHg | 1.44 (1.14 - 1.83) | 1.10 (0.94 - 1.27) | 0.90 (0.76 - 1.07) | 0.69 (0.55 - 0.86) |
|  | 51 - 55mmHg | 1.10 (0.85 - 1.43) | 0.86 (0.71 - 1.06) | 0.68 (0.55 - 0.83) | 0.74 (0.53 - 1.04) |
|  | > 55mmHg | 0.96 (0.75 - 1.22) | 1.00 (0.84 - 1.19) | 0.61 (0.48 - 0.77) | 0.40 (0.23 - 0.70) |

**Table 4**

|  | | PaO_2_ | | |
| --- | --- | --- | --- | --- |
|  |  | < 60mmHg  AOR (95% CI) | 60 - 100mmHg  AOR (95% CI) | > 100mmHg  AOR (95% CI) |
| PaCO_2_ | ≤ 35mmHg | 1.44 (1.11 - 1.85) | 1.08 (0.92 - 1.27) | 1.70 (1.38 - 2.11) |
|  | 36 - 45mmHg | 1.34 (1.10 - 1.65) | 1.15 (0.98 - 1.35) | 1 (reference category) |
|  | 46 - 50mmHg | 1.75 (1.17 - 2.62) | 1.32 (0.91 - 1.92) | 0.61 (0.41 - 0.89) |
|  | 51 - 55mmHg | 1.43 (0.86 - 2.37) | 1.26 (0.79 - 2.01) | 0.53 (0.33 - 0.86) |
|  | > 55mmHg | 1.96 (1.19 - 3.22) | 1.53 (0.96 - 2.44) | 0.42 (0.26 - 0.68) |

**Table 5**

| Variable | Incidence | **Hospital mortality, n (%)** | **Unadjusted Odds ratio for mortality (95% CI)** | **Adjusted Odds ratio for mortality (95% CI)** |
| --- | --- | --- | --- | --- |
| PaO_2_  < 60mmHg  60 - 100mmHg  101-200mmHg  201 – 300mmHg  > 300mmHg | 4,135 / 23,625 (17.5%)  17,480 / 23,625 (74.0%)  1,939 / 23,625 (8.2%)  40 / 23,625 (0.2%)  31 / 23,625 (0.1%) | 2,704 / 4,135 (65.4%)  10,187 / 17,480 (58.3%)  1,045 / 1,939 (53.9%)  19 / 40 (47.5%)  21 / 31 (67.7%) | 1.61 (1.45 -1.80), P < 0.001  1.19 (1.09 - 1.31), P < 0.001  1 (reference category)  0.77 (0.41 - 1.45), P = 0.423  1.80 (0.84 - 3.84), P = 0.130 | 1.34 (1.09 - 1.65), P = 0.005  1.15 (0.98 - 1.36), P = 0.094  1 (reference category)  0.58 (0.22 - 1.55), P = 0.282  2.95 (0.50 - 17.46), P = 0.234 |

**Table 6**

|  | | PaO_2_ | | | | |
| --- | --- | --- | --- | --- | --- | --- |
|  |  | < 60mmHg  AOR (95% CI) | 60 - 100mmHg  AOR (95% CI) | 101 - 200mmHg  AOR (95% CI) | 201 - 300mmHg  AOR (95% CI) | > 300mmHg  AOR (95% CI) |
| PaCO_2_ | ≤ 35mmHg | 1.45 (1.12 -1.87) | 1.09 (0.92 - 1.29) | 1.69 (1.36 - 2.09) | 0.58 (0.22 - 1.55)^a^ | 1.26 (0.22 - 7.11) |
|  | 36 - 45mmHg | 1.34 (1.09 - 1.65) | 1.15 (0.98 - 1.36) | 1 (reference category) | 0.58 (0.22 - 1.55)^a^ | 2.95 (0.50 - 17.46) |
|  | 46 - 50mmHg | 1.77 (1.18 - 2.67) | 1.34 (0.92 - 1.96) | 0.60 (0.40 - 0.89) | 0.88 (0.27 - 2.81) | 1.99 (0.17 - 23.10) |
|  | 51 - 55mmHg | 1.48 (0.91 - 2.40) | 1.30 (0.83 - 2.04) | 0.52 (0.32 - 0.82) | 1.98 (0.13 - 29.27) | 1.36 (0.16 - 11.44) |
|  | > 55mmHg | 1.96 (1.18 - 3.27) | 1.54 (0.94 - 2.51) | 0.42 (0.25 - 0.70) | 0.78 (0.11 - 5.67) | 1.37 (0.19 - 10.07) |

**Table 7**

| Variable | Incidence | **Hospital mortality, n (%)** | **Unadjusted Odds ratio for hospital mortality (95% CI)** | **Adjusted Odds ratio for hospital mortality (95% CI)** |
| --- | --- | --- | --- | --- |
| PaO_2_/FiO_2_^a^  ≤ 100mmHg  101-200mmHg  201 – 300mmHg  > 300mmHg | 5,112 / 28,881 (17.7%)  10,133 / 28,881 (35.1%)  8,266 / 28,881 (28.6%)  5,370 / 28,881 (5,370) | 4,021 / 5,112 (78.7%)  6,933 / 10,133 (68.4%)  4,940 / 8,266 (59.8%)  2,916 / 5,370 (54.3%) | 3.10 (2.85 – 3.38), P < 0.001  1.82 (1.70 – 1.95), P < 0.001  1.25 (1.17 – 1.34), P < 0.001  1 (reference category) | 1.94 (1.62 – 2.31), P < 0.001  1.67 (1.49 – 1.88), P < 0.001  1.36 (1.21 – 1.54), P < 0.001  1 (reference category) |
| PaO_2_^b^  Hypoxemia  Normoxia  Hyperoxemia | 5,389 / 28,883 (18.7%)  20,404 / 28,883 (70.6%)  3,090 / 28,883 (10.7%) | 3,992 / 5,389 (72.8%)  12,859 / 20,404 (63.0%)  2,031 / 3,090 (65.7%) | 1.39 (1.27 – 1.53), P < 0.001  0.89 (.082 – 0.96), P = 0.004  1 (reference category) | 1.33 (1.09 – 1.61), P = 0.004  1.11 (0.95 – 1.30), P = 0.184  1 (reference category) |
| PaCO_2_^a^  ≤35mmHg  36 – 45mmHg  46 – 50mmHg  51 – 55mmHg  >55mmHg | 6,603 / 28,883 (22.9%)  11,491 / 28,883 (39.8%)  4,158 / 28,883 (14.4%)  2,494 / 28,883 (8.6%)  4,137 / 28,883 (14.3%) | 4,579 / 6,603 (69.3%)  6,993 / 11,491 (60.9%)  2,556 / 4,158 (61.5%)  1,579 / 2,494 (63.3%)  3,105 / 4,137 (75.05%) | 1.46 (1.36 – 1.55), P < 0.001  1 (reference category)  1.03 (0.95 – 1.10), P = 0.486  1.11 (1.01 – 1.21), P = 0.023  1.94 (1.79 – 2.10), P < 0.001 | 1.95 (1.67 – 2.27), P < 0.001  1 (reference category)  0.72 (0.58 – 0.88), P = 0.001  0.73 (0.52 – 1.03), P = 0.070  0.50 (0.29 – 0.86), P = 0.012 |

**Table 8**

|  | | PaO_2_/FiO_2_ | | | |
| --- | --- | --- | --- | --- | --- |
|  |  | ≤ 100mmHg  AOR (95% CI) | 101 - 200mmHg  AOR (95% CI) | 201 - 300mmHg  AOR (95% CI) | > 300mmHg  AOR (95% CI) |
| PaCO_2_ | ≤ 35mmHg | 3.10 (2.39 - 4.03) | 2.72 (2.31 - 3.20) | 2.19 (1.89 - 2.54) | 1.95 (1.67- 2.27) |
|  | 36 - 45mmHg | 1.94 (1.62 - 2.31) | 1.67 (1.49 - 1.88) | 1.36 (1.21 - 1.54) | 1 (reference category) |
|  | 46 - 50mmHg | 1.55 (1.23 - 1.96) | 1.11 (0.95 - 1.29) | 0.91 (0.76 - 1.08) | 0.72 (0.58 - 0.88) |
|  | 51 - 55mmHg | 1.19 (0.93 - 1.53) | 0.85 (0.69 - 1.04) | 0.68 (0.55 - 0.83) | 0.73 (0.52 - 1.03) |
|  | > 55mmHg | 1.00 (0.79 - 1.26) | 0.96 (0.81 - 1.14) | 0.60 (0.47 - 0.77) | 0.50 (0.29 - 0.86) |

**Table 9**

|  | | PaO_2_ | | |
| --- | --- | --- | --- | --- |
|  |  | < 60mmHg  AOR (95% CI) | 60 - 100mmHg  AOR (95% CI) | > 100mmHg  AOR (95% CI) |
| PaCO_2_ | ≤ 35mmHg | 1.34 (1.05 - 1.71) | 0.96 (0.82 - 1.12) | 1.86 (1.52- 2.27) |
|  | 36 - 45mmHg | 1.33 (1.09 - 1.61) | 1.11 (0.95 - 1.30) | 1 (reference category) |
|  | 46 - 50mmHg | 1.52 (1.05 - 2.21) | 1.16 (0.82 - 1.63) | 0.67 (0.47 - 0.97) |
|  | 51 - 55mmHg | 1.26 (0.80 - 1.99) | 1.09 (0.72 - 1.65) | 0.59 (0.38 - 0.90) |
|  | > 55mmHg | 1.39 (0.88 - 2.21) | 1.07 (0.69 - 1.65) | 0.56 (0.35 - 0.90) |

**Table 10**

| Variable | Incidence | **Hospital mortality, n (%)** | **Unadjusted Odds ratio for mortality (95% CI)** | **Adjusted Odds ratio for mortality (95% CI)** |
| --- | --- | --- | --- | --- |
| PaO_2_  < 60mmHg  60 - 100mmHg  101-200mmHg  201 – 300mmHg  > 300mmHg | 5,389 / 28.883 (18.7%)  20,404 / 28,883 (70.6%)  2,861 / 28,883 (9.9%)  147 / 28,883 (0.5%)  82 / 28,883 (0.3%) | 3,922 / 5,389 (72.8%)  12,859 / 20,404 (63.0%)  1,848 / 2,861 (64.6%)  117 / 141 (79.6%)  66 / 82 (80.5%) | 1.47 (1.33 – 1.61), P < 0.001  0.93 (0.86 – 1.01), P = 1.03  1 (reference category)  2.14 (1.42 – 3.22), P < 0.001  2.26 (1.30 – 3.93), P = 0.004 | 1.34 (1.10 – 1.63), P = 0.004  1.12 (0.96 – 1.32), P = 0.158  1 (reference category)  0.98 (0.42 – 2.28), P = 0.963  4.45 (1.12 – 17.69), P = 0.034 |

**Table 11**

|  | | PaO_2_ | | | | |
| --- | --- | --- | --- | --- | --- | --- |
|  |  | < 60mmHg  AOR (95% CI) | 60 - 100mmHg  AOR (95% CI) | 101 - 200mmHg  AOR (95% CI) | 201 - 300mmHg  AOR (95% CI) | > 300mmHg  AOR (95% CI) |
| PaCO_2_ | ≤ 35mmHg | 1.37 (1.07 - 1.75) | 0.98 (0.84 - 1.15) | 1.84 (1.49 - 2.27) | 0.98 (0.42 - 2.28)^a^ | 1.47 (0.27 - 7.88) |
|  | 36 - 45mmHg | 1.34 (1.10 - 1.63) | 1.12 (0.96 - 1.32) | 1.00 | 0.98 (0.42 - 2.28)^a^ | 4.45 (1.12 - 17.69) |
|  | 46 - 50mmHg | 1.53 (1.05 - 2.25) | 1.16 (0.82 - 1.66) | 0.68 (0.47 - 0.98) | 0.88 (0.34 - 2.29) | 1.75 (0.18 - 16.79) |
|  | 51 - 55mmHg | 1.32 (0.84 - 2.05) | 1.14 (0.76 - 1.70) | 0.57 (0.38 - 0.86) | 2.94 (0.32 - 27.39) | 1.27 (0.16 - 10.18) |
|  | > 55mmHg | 1.38 (0.87 - 2.21) | 1.06 (0.68 - 1.65) | 0.57 (0.35 - 0.93) | 0.64 (0.14 - 2.91) | 1.24 (0.20 - 7.90) |
